# Selective Interactions of *Valeriana officinalis* Extracts and Valerenic Acid with [^3^H]Glutamate Binding to Rat Synaptic Membranes

**DOI:** 10.1155/2011/403591

**Published:** 2011-04-26

**Authors:** Lisa M. Del Valle-Mojica, Yoshira M. Ayala-Marín, Carmen M. Ortiz-Sanchez, Bianca A. Torres-Hernández, Safa Abdalla-Mukhaimer, José G. Ortiz

**Affiliations:** ^1^Department of Pharmacology and Toxicology, School of Medicine, University of Puerto Rico-Medical Sciences Campus, P.O. Box 365067, San Juan 00936-5067, Puerto Rico; ^2^Department of Biology, Natural Sciences Faculty, University of Puerto Rico Humacao Campus, Humacao 00791-4300, Puerto Rico; ^3^Department of Chemistry, Natural Sciences Faculty, University of Puerto Rico Cayey Campus, Cayey 00736-9997, Puerto Rico

## Abstract

Although GABA neurotransmission has been suggested as a mechanism for *Valeriana officinalis* effects, CNS depression can also be evoked by inhibition of ionotropic (iGluR) and metabotropic glutamate receptors (mGluR). In this study, we examined if aqueous valerian extract interacted with glutamatergic receptors. Freshly prepared aqueous valerian extract was incubated with rat cortical synaptic membranes in presence of 20 nM [^3^H]Glutamate. Aqueous valerian extract increased [^3^H]Glutamate binding from 1 × 10^−7^ to 1 × 10^−3^ mg/mL. In the presence of (2S,1′S,2′S)-2-(Carboxycyclopropyl)glycine (LCCG-I) and (2*S*,2′*R*,3′*R*)-2-(2′,3′-Dicarboxycyclopropyl)glycine (DCG-IV), Group II mGluR agents, valerian extract markedly decreased [^3^H]Glutamate binding, while (2*S*)-2-amino-3-(3,5-dioxo-1,2,4-oxadiazolidin-2-yl) propanoic acid) (quisqualic acid, QA), Group I mGluR agonist, increased [^3^H]Glutamate binding. At 0.05 mg/mL aqueous valerian extract specifically interacted with kainic acid NMDA and AMPA receptors. Valerenic acid, a marker compound for *Valeriana officinalis*, increased the [^3^H]Glutamate binding after 1.6 × 10^−2^ mg/mL, and at 0.008 mg/mL it interacted only with QA (Group I mGluR). The selective interactions of valerian extract and valerenic acid with Group I and Group II mGluR may represent an alternative explanation for the anxiolytic properties of this plant.

## 1. Introduction

*Valeriana officinalis L., s.l.* (Valerianaceae family) is a medicinal plant used in complementary and alternative medicine for its sedative and anxiolytic properties [1, 2]. Valerian's effects on the central nervous system have been well documented and attributed to many of it active compounds: valepotriates, baldrinals, valerenic acid, valerenal and valeranone, and other constituents in the essential oils [1, 3–8]. Albeit the anxiolytic properties of valerian have been demonstrated in animals [9, 10], there are no sufficient studies in humans [6]. Consequently, the therapeutic properties of *Valeriana officinalis* have yet to be conclusively demonstrated [6, 9, 11].

“Anxiety and stress-related diseases are a group of disorders that have in common excessive or inappropriate brain excitability within crucial brain circuits” [12]. The actions of glutamate, the major excitatory neurotransmitter, are mediated by two types of receptors: (1) ionotropic receptors (iGluR): NMDA, AMPA, and kainate receptors and (2) metabotropic receptors (mGluR) which are comprised of three groups (I, II, and III). Decreasing excitatory neurotransmission in the CNS by modulating glutamate receptors is an alternative approach to produce anxiolysis and sedation.

The present study investigated the interaction of *Valeriana officinalis* aqueous extract with the glutamatergic receptors. We also examined the interaction of valerenic acid and isoborneol, two constituents present in the extracts. To accomplish this objective, receptor-binding assays were performed using rat cortical synaptic membranes in the presence of fresh valerian extracts and both types of glutamate receptor ligands.

## 2. Materials and Methods

### 2.1. Chemicals

Valerenic acid (99.7% purity-Lot no. 22150-1800) and isoborneol (96.6% purity-Lot no. 09195-102) were purchased from Chromadex, Irvine, CA. L-[3,4-^3^H]-Glutamic acid (49.9 Ci/mmol) was obtained from PerkinElmer Life and Analytical Sciences, Inc. (Shelton, CT). AMPA (RS)-a-Amino-3-hydroxy-5-methyl-4-isoxazolepropionic acid), NMDA (N-methyl-D-aspartic acid), kainic acid, quisqualic acid (2*S*)-2-amino-3-(3,5-dioxo-1,2,4-oxadiazolidin-2-yl) propanoic acid), LCCG-I (2S,1′S,2′S)-2-(Carboxycyclopropyl)glycine, EGLU ((2S)-a-Ethylglutamic acid), spaglumic acid (N-Acetyl-L-aspartyl-L-glutamic acid) and L-AP4 (L-(+)-2-Amino-4-phosphonobutyric acid) were obtained from Tocris Bioscience (Ellisville, MO). UniverSol ES was obtained from MP Biomedicals (Solon, OH). All other reagents were obtained from Sigma-Aldrich Co. (St. Louis, MO).

### 2.2. Valerian Extracts

*Valeriana officinalis L., s.l.* dry powdered roots (Lot. 1111H-OUP), harvested in 2004 and organically grown/certified, were obtained from Pacific Botanicals (LLC Grants Pass, Oregon). Valerian was extracted in ultra pure water, typically (1 : 10 w/v) at ~23°C and stirred for 1 hour. Aliquots were centrifuged at *6,700 g* to remove particulates.

### 2.3. Valerenic Acid

A 10 mM stock solution was prepared using EtOH 95%. The dilutions used for the assays were freshly prepared with 50 mmTris-HCl/100 mM KCl buffer before each experiment.

### 2.4. Isoborneol

A 65 mM stock solution was prepared using EtOH 95%. The dilutions used for the assays were freshly prepared with EtOH 70% before each experiment.

### 2.5. Cerebral Cortex Synaptic Membranes

Cerebral cortex synaptic membranes were purchased from Analytical Biological Services, Inc. (Wilmington, DE). They report that these were prepared as follows: female rats of approximately two months of age were decapitated and the brain promptly removed. The cortex was dissected and homogenized (1 : 10 w/v) in ice-cold 10 mM TRIS-HCl buffer pH 7.4. The homogenate was centrifuged twice at *2,500 g* for 10 min. The resulting supernatant was centrifuged at *12,500 g *for 20 min. The pellet was washed twice with ice-cold 10 mM TRIS-HCl buffer pH 7.4 (1 : 10 w/v) and centrifuged at *12,500 g* for 20 min. The pellet (synaptical membrane, P2) was resuspended in 10 mM TRIS-HCl buffer pH 7.4 and freeze thawed at least three times before been stored at −80°C until used. Protein concentration was determined using the Bradford assay [13] using bovine serum albumin (BSA) as reference standard.

### 2.6. [^3^H]Glutamate Binding

Receptor-binding competition assays were done using cerebral cortex synaptic membranes from Analytical Biological Services, Inc. (Wilmington, DE). The reaction was initiated by the addition of tissue (100 *μ*g protein) to tubes containing 1 mM of different iGluR and mGluR ligands and 20 nM [^3^H]Glutamic acid in a final volume of 500 *μ*L of 50 mM Tris HCl/100 mM KCl buffer, pH 7.4. The nonspecific binding was determined in the presence of 1 mM nonradioactive glutamate. All samples were incubated on ice (0–4°C) for 40 minutes. The assay was stopped by centrifugation for 30 min at *6,700 g;* the supernatant was extracted, and the pellet was washed two times with 1 mL of ice-cold buffer. The pellet was resuspended in 500 *μ*l of buffer. Radioactivity of the samples was quantified in a Beckman LS 6500 Multipurpose Scintillation Counter with 1 mL of UniverSol ES scintillation cocktail. Results are shown as percentage of total binding (SEM).

### 2.7. [^3^H]Glutamate Displacement Curves

Different concentrations of freshly prepared aqueous valerian extracts (4 pg/mL–12 mg/mL), valerenic acid (30 ng/mL–2 mg/mL), and Isoborneol (5 ng/mL–2 mg/mL) were incubated with rat cortical membranes in presence of 20 nM [^3^H]Glutamate.

### 2.8. Statistical Analysis

Data were expressed as mean values ± the standard error of the mean (SEM) of at least three experiments. The differences between the experimental groups were tested for significance using one way analysis of variance followed by Tukey-Kramer multiple comparisons test, with *P* < .05. Statistics for the experimental group versus total binding were not shown for clarity.

## 3. Results

### 3.1. [^3^H]Glutamate Displacement Curves for Valerian and Its Constituents

**Figure 1:**
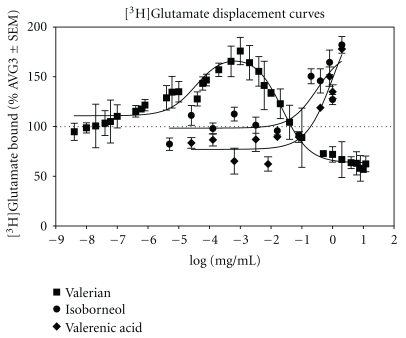
Effects of different valerian, valerenic acid, and isoborneol concentrations on [^3^H]Glutamate binding. Synaptic membranes were incubated with different concentrations of aqueous valerian extract, valerenic acid, and isoborneol in presence of 20 nM [^3^H]Glutamate. The maximum glutamate binding was 160% for valerian extract concentration of 0.001 mg/mL. The [^3^H]Glutamate displacement curves in presence of valerenic acid and isoborneol were very different from the curve in presence of valerian. Each point represents at least three independent assays.

The effect of different valerian extract concentrations, valerenic acid and isoborneol in presence of [^3^H]Glutamate were dose dependent, as shown in Figure 1. Valerian increased [^3^H]Glutamate binding from 8 × 10^−7^–1 × 10^−1^ mg/mL reaching a maximum binding of 160% at 1 × 10^−3^ mg/mL. At higher valerian concentrations, greater than 1 × 10^−3^ mg/mL, there is a sudden decrease in [^3^H]Glutamate binding, reaching 50% at 12 mg/mL. In contrast, valerenic acid and isoborneol increased [^3^H]Glutamate binding at higher concentrations (1.6 × 10^−2^ to 2 mg/mL). 

### 3.2. *In Vitro* Receptor Selectivity Studies

**Figure 2:**
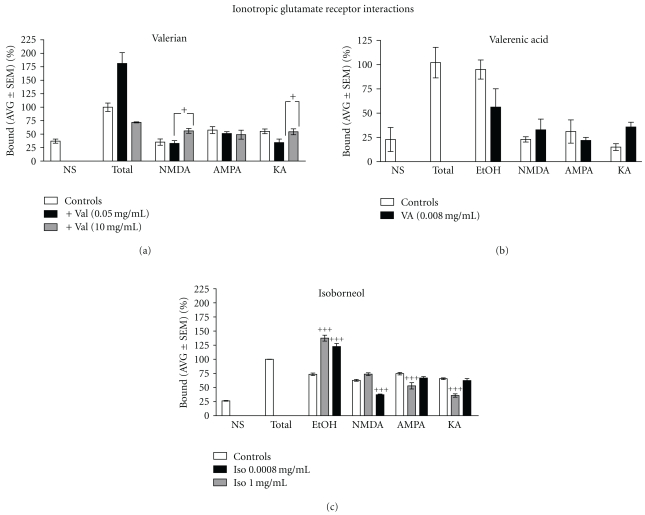
Effects of valerian extracts, valerenic acid, and isoborneol on ionotropic glutamate receptors (iGluR). (a) Valerian extract at 0.05 mg/ml in presence of 1 mM kainic acid (KA) decreased the glutamate binding significantly. This effect was not seen with NMDA or AMPA. High concentrations of valerian extract (10 mg/ml) in presence of NMDA (1 mM) significantly decreased the glutamate binding. Neither AMPA nor KA in presence of higher valerian extract concentrations affected the binding. (b) Valerenic acid at 0.008 mg/mL, in presence of 1 mM NMDA, kainic acid (KA) and AMPA, did not affect the glutamate binding. (c) Isoborneol at 0.0008 mg/mL, in presence of 1 mM NMDA, significantly decreased the glutamate binding. At higher concentration (1 mg/mL), isoborneol markedly decreased the glutamate binding in presence of AMPA and KA. **^+^** *agonist versus agonist *+* (valerian, valerenic acid*,* or isoborneol)*, *P* < .05; ^++^ *P* < .01; ^+++^ *P* < .001.

Having shown that *Valeriana officinalis* and it constituents interacted with [^3^H]Glutamate binding, we examined the type of glutamatergic receptor involved. For this purpose, binding assays were done in presence of different types of ionotropic (iGluR) and metabotropic glutamate receptor (mGluR) ligands.  Figure 2(a) shows that Valerian extract at 0.05 mg/ml increased [^3^H]Glutamate binding by 81%. A decrease (21%) in [^3^H]Glutamate binding is observed with KA (1 mM) but not with NMDA or AMPA. In the presence of NMDA (1 mM), higher valerian extract concentration (10 mg/mL) increased the [^3^H]Glutamate binding significantly (20%). Valerian (10 mg/mL) did not interact with AMPA or KA. 

The same assay was performed with 0.008 mg/mL of valerenic acid, and it showed (Figure 2(b)) that valerenic acid decreased the [^3^H]Glutamate binding more than 40%. However, valerenic acid did not change the effects of iGluR ligands on [^3^H]Glutamate binding. In contrast, Figure 2(c) shows that isoborneol (0.0008 mg/mL) only interacted with NMDA. At higher concentrations (1 mg/mL) isoborneol interacted with AMPA and KA.

**Figure 3:**
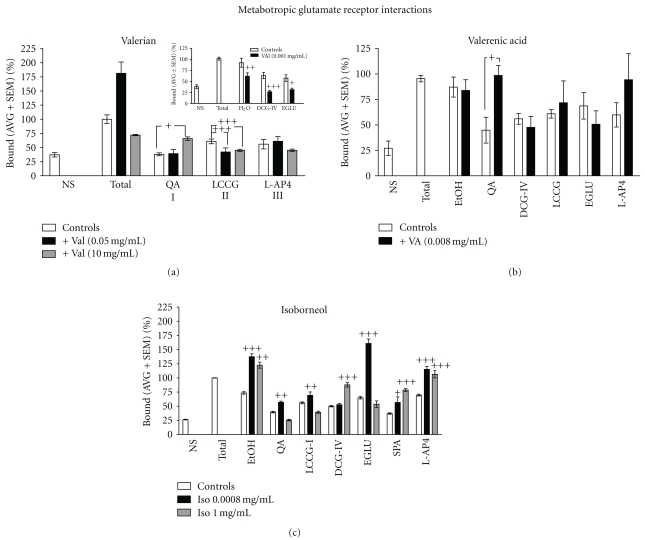
Interactions of valerian extracts, valerenic acid, and isoborneol on metabotropic glutamate receptors (mGluR). (a) At lower concentrations of valerian extract (0.05 mg/mL) in presence of LCCG-I, a Group II metabotropic glutamate receptor agonist, there was a marked decrease in the binding. This effect was not seen with QA and L-AP4. Valerian extracts at 10 mg/mL, in presence of both QA and LCCG-I, significantly decreased the glutamate binding. ((a)-Insert) A marked decrease in the [^3^H]Glutamate binding was observed when valerian extracts (0.001 mg/mL) were in the presence of DCG-IV and EGLU, a Group II metabotropic glutamate receptor agonist and antagonist, respectively. (b) Valerenic acid concentration at 0.008 mg/mL, in the presence of QA, a Group I metabotropic glutamate receptor agonist, produced a significant increase in the binding. No effects were observed in presence of DCG-IV, LCCG-I, EGLU, and L-AP4. (c) Isoborneol, at 0.0008 and 1 mg/mL increased [^3^H]Glutamate binding by 49% and 64%, respectively. Isoborneol in the presence of mGluR ligands, indiscriminately interacted with all of them. At 0.0008 mg/mL, isoborneol interacted with QA and markedly decreased (14%) [^3^H]Glutamate binding. In addition, isoborneol interacted with all the mGluR agonists (LCCG-I, DCG-IV, and spaglumic) and antagonist (EGLU). Isoborneol also strongly interacted with L-AP4 at both concentrations (0.0008 mg/mL and 1 mg/mL) and resulted in an increased [^3^H]Glutamate binding (37% and 46%, resp.). *^+^ agonist versus agonist* + *(valerian, valerenic acid*,* or isoborneol), P* < .05; ^++^ *P* < .01; ^+++^ *P* < .001.

As shown in Figure 3(a), valerian extract (0.05 mg/mL) increased [^3^H]Glutamate binding by 81%, while at 10 mg/mL, it decreased the binding by 28%. Valerian extract (0.05 mg/mL) in presence of LCCG-I, a Group II metabotropic glutamate receptor agonist, decreased (19%) [^3^H]Glutamate binding. This effect was not observed with QA or L-AP4. In contrast, at 10 mg/mL, the valerian extract, in presence of QA, increased the glutamate binding (28%), while in presence of LCCG-I, it decreased [^3^H]Glutamate binding by 16%. A detailed evaluation of Group II mGluR-Valerian interaction (Figure 3(a)-Insert) confirmed a marked decrease in the [^3^H]Glutamate binding when valerian extracts (0.001 mg/mL) were in presence of DCG-IV (28%) and EGLU (38%), a highly selective Group II metabotropic glutamate receptor agonist and antagonist, respectively.

In presence of QA, valerenic acid (0.008 mg/mL) significantly increased the [^3^H]Glutamate binding by 54% as shown in Figure 3(b). Similarly, isoborneol at 0.0008 and 1 mg/mL increased [^3^H]Glutamate binding by 49% and 64%, respectively (Figure 3(c)). Interestingly, isoborneol indiscriminately interacted with all the mGluR ligands, and at 0.0008 mg/mL, isoborneol interacted with QA and markedly decreased (14%) [^3^H]Glutamate binding. In addition, isoborneol interacted with all the mGluR agonists (LCCG-I, DCG-IV and spaglumic) and antagonist (EGLU). Isoborneol also strongly interacted with L-AP4 at both concentrations (0.0008 mg/mL and 1 mg/mL) and resulted in an increased [^3^H]Glutamate binding (37% and 46%, resp.).

## 4. Discussion

Among 150 potentially active phytochemicals, constituents identified in the Valerian's roots are (1) iridoid valepotriates (0.5%–2.0%): valtrate, isovaltrate, didrovaltrate, acevaltrate and others and (2) volatile essential oil (0.2%–2.8%) divided in (a) monoterpenes: borneol and bornyl acetate and (b) sesquiterpenes: valerenic, valeric, isovaleric and acetoxyvalerenic acids: valerenal, valeranone, and kessyl glycol [14, 15]. Some sesquiterpenes, such valerenic acid, influence serotonin and noradrenaline levels, reduce locomotion, and anxiety, and increase pentobarbital sleeping time of mice [16, 17]. Similarly, other constituents, such as valepotriates potentiate hexobarbital anesthesia, suppress aggression, have anticonvulsant effects against pentylenetetrazole- and strychnine-induced seizures, increase thiopental sleeping time, reduce motility, and have dose-dependent sedative effects [17–19]. In addition, valepotriates and their decomposition products, baldrinals and homobaldrinals, reduced the total HAM-A (Hamilton anxiety scale) in a small clinical trial [20] and increased the time spent in open arms when elevated plus maze test was performed in rats [19]. Therefore, it is most likely that valerian's pharmacological properties are due to the additive effects of multiple active constituents and not one alone [21]. 

Most of the Valerian effects described above are consistent with the enhancement of GABAa-mediated transmission [7, 22–24]. Cavadas and colleagues showed that valerian extracts bind to GABA_a_ receptors [4], while other researchers found that valerian is a partial agonist of the 5-HT(5a) receptor [8] and promotes cell proliferation in the hippocampus of “depressive” rats [25]. Moreover, recent studies suggest that different components within the valerian extract mediate the activation of adenosine receptors [26, 27]. Indeed, many studies have been done to determine the interaction of valerian with different receptors, but none of them examined valerian-glutamate receptor interaction.

Our [^3^H]Glutamate displacement curve in presence of *Valeriana officinalis* demonstrated that valerian extracts increase glutamate binding from 8 × 10^−7^ to 1 × 10^−1^ mg/mL reaching a maximum ^3^[H]Glutamate binding at 1 × 10^−3^ mg/mL (160%). Therefore, at these physiological attainable concentrations (8 × 10^−7^–1 × 10^−1^ mg/mL) valerian potentiates ^3^[H]Glutamate binding. In contrast, different findings were obtained for valerenic acid and isoborneol. The [^3^H]Glutamate displacement curves and the receptor selectivity obtained for valerian and its constituents, for instance, are very different from each other, suggesting that these constituents are not the compounds responsible for the increase in ^3^[H]Glutamate binding observed with valerian. 

The glutamatergic system plays an important role in anxiety pathogenesis [28]. It is suggested that the physiological and behavioral responses associated with anxiety are regulated by a balance between the inhibition produced by GABA and the excitation caused by glutamate. Treatments used to decrease excitability in neurons from the amygdala are achieved by increasing GABA neurotransmission. Alternatively, treatments to decrease the excitability can be obtained by decreasing the excitatory glutamatergic transmission. Therefore, decreasing excitatory neurotransmission in the CNS, by modulating the response produced by the glutamatergic receptors, is an alternative approach to produce anxiolysis and sedation. The glutamate receptors found in the amygdala produce excitatory and inhibitory actions, and the degree of ionotropic and metabotropic activation is an important factor to determine the amygdala cell excitability. Thus, the modulation of glutamate actions mediated by iGluR and mGluR represent a feasible alternative to treat anxiety states [12, 29].

Compounds that decrease glutamatergic transmission via blockade of NMDA have been reported to produce anxiolytic and antidepressant like actions in animal tests and models [30, 31]. Our group demonstrated that valerian extract had modest inhibitory effects on [^3^H]MK-801 binding, an indicator of NMDA-valerian interaction [32]. In 2004, Malva and colleagues reported that by decreasing neuronal network excitability through AMPA permeable Ca^2+^ receptors, valerian preparations could contribute to neuroprotection and may be of therapeutic use in preventing glutamate-mediated degeneration related to aging or neurodegenerative disorders [33]. Now, our study confirms that in the presence of iGluR agonists, valerian extracts interact with KA and NMDA (0.05 mg/mL and 10 mg/mL, resp.). While isoborneol exhibited significant interactions with all iGluR, valerenic acid did not interact with iGluR agonists. 

Many studies suggest that metabotropic glutamate receptors are involved in anxiety [12, 34–36]. For this reason, we performed receptor-binding assays with valerian extracts in presence of different types of metabotropic glutamate receptors. In this study, we demonstrated that valerian extracts, in presence of mGluR ligands, exhibited significant interaction with QA (Group I mGluR) and LCCG-I (Group II mGluR). Valerenic acid selectively interacts with QA. However, isoborneol interacts with all mGluR receptors. Our results clearly demonstrated that *Valeriana offiicinalis* and it constituents (valerenic acid and isoborneol) interact with Group I and II mGluR, which supports previous studies showing the role of Group I and II mGluR in anxiety [35, 37].

The pharmacological effects of *Valeriana officinalis* extracts in [^3^H]Glutamate binding are not conventional and easy to understand. They could reflect complex interactions (agonist/antagonist) or synergism occurring in the complex mixture of phytochemical constituents. The biphasic [^3^H]Glutamate displacement curve obtained in presence of valerian represents an example of the interactions that occur in an extract which could not be evident when single constituents are studied in isolation. This study exclusively used receptor-binding assays to determine ligand-receptor interactions, but functionalities studies are being conducted to characterize them.

Our results confirm the hypothesis of valerian interaction with the glutamatergic receptors, suggesting a possible mechanism by which valerian extracts produce their effects. Val-mGluR interactions may represent a novel alternative for the treatment of anxiety. Further studies with fractions of valerian extract should be done to evaluate the interactions between a group of compounds and their effects on receptor-binding selectivity.

## Abbreviations

AMPA: (*α*-amino-3-hydroxyl-5-methylisoxazole-4-propionic acid)
CAM: Complementary alternative medicine
CNS: Central nervous system
DCG-IV: (2*S*,2′*R*,3′*R*)-2-(2′,3′-Dicarboxycyclopropyl)glycine
EGLU: (2S)-a-Ethylglutamic acid)
Group II mGluR: Metabotropic glutamate receptors (2/3)
Group I mGluR: Metabotropic glutamate receptor (1/5)
iGluR: Ionotropic glutamate receptor
KA: Kainic acid
L-AP4: L-(+)-2-Amino-4-phosphonobutyric acid
LCCG-I: (2S,1′S,2′S)-2-(Carboxycyclopropyl)glycine
mGluR: Metabotropic glutamate receptor
NCCAM: National Center of Complementary Alternative Medicine
NMDA: N-methyl-D-aspartic acid
QA: quisqualic acid: (2*S*)-2-amino-3-(3,5-dioxo-1,2,4-oxadiazolidin-2-yl) propanoic acid)
Spaglumic acid: (N-Acetyl-L-aspartyl-L-glutamic acid).
